# Metal–Support Interactions in Single-Atom Catalysts for Electrochemical CO_2_ Reduction

**DOI:** 10.3390/nano16020103

**Published:** 2026-01-13

**Authors:** Alexandra Mansilla-Roux, Mayra Anabel Lara-Angulo, Juan Carlos Serrano-Ruiz

**Affiliations:** Materials and Sustainability Group, Department of Engineering, Universidad Loyola Andalucía, Avda. de las Universidades s/n, 41704 Dos Hermanas, Seville, Spain

**Keywords:** CO_2_ electroreduction, single-atom catalysts, metal–support interactions, faradaic efficiency, operando characterization, rational catalyst design

## Abstract

Electrochemical CO_2_ reduction (CO_2_RR) is a promising route to transform a major greenhouse gas into value-added fuels and chemicals. However, its deployment is still hindered by the sluggish activation of CO_2_, poor selectivity toward multielectron products, and competition with the hydrogen evolution reaction (HER). Single-atom catalysts (SACs) have emerged as powerful materials to address these challenges because they combine maximal metal utilization with well-defined coordination environments whose electronic structure can be precisely tuned through metal–support interactions. This minireview summarizes current understanding of how structural, electronic, and chemical features of SAC supports (e.g., porosity, heteroatom doping, vacancies, and surface functionalization) govern the adsorption and conversion of key CO_2_RR intermediates and thus control product distributions from CO to CH_4_, CH_3_OH and C_2_^+^ species. Particular emphasis is placed on selectivity descriptors (e.g., coordination number, d-band position, binding energies of *COOH and *OCHO) and on rational design strategies that exploit curvature, microenvironment engineering, and electronic metal–support interactions to direct the reaction along desired pathways. Representative SAC systems based primarily on N-doped carbons, complemented by selected examples on oxides and MXenes are discussed in terms of Faradaic efficiency (FE), current density and operational stability under practically relevant conditions. Finally, the review highlights remaining bottlenecks and outlines future directions, including operando spectroscopy and data-driven analysis of dynamic single-site ensembles, machine-learning-assisted DFT screening, scalable mechanochemical synthesis, and integration of SACs into industrially viable electrolyzers for carbon-neutral chemical production.

## 1. Introduction

Anthropogenic CO_2_ emissions resulting from fossil fuel combustion continue to rise, intensifying global warming and destabilizing ecological systems [1]. Although carbon capture and storage (CCS) has been proposed as a potential solution, its widespread application remains limited [2]. In this context, carbon valorization has emerged as a complementary strategy for decreasing net CO_2_ emissions while also converting waste carbon into valuable chemicals, fuels, and materials [3]. Among the CO_2_ valorization technologies available today, electrochemical reduction (CO_2_RR) holds great promise since: (i) it allows to carry out the reduction process at mild conditions (ii) it does not require external H_2_ since water can be used as the source of both electrons and H^+^ required for the reduction in CO_2_ and (iii) and can make use of renewable wind- or solar-derived electricity, thereby reducing the carbon footprint of the conversion process [4,5].

Despite this potential, CO_2_RR is currently limited by three main aspects. First, there is a selectivity issue. Thus, CO_2_RR is a rather complex process that can involve multiple proton and electron transfer steps. As a result, a large variety of compounds including C_1_ (e.g., formic acid, CO, methanol, methane) and C_2_ (e.g., ethylene and ethanol) species can be formed over a narrow range of equilibrium potentials, making it difficult to control the selectivity during the process [5,6]. In this sense, while the production of two-electron products such as formic acid and CO is relatively straightforward, the selective generation of more appealing and higher value multiple electron products such methanol, methane, and C_2_ products is normally carried out with low faradaic efficiency (FE) [6,7]. Furthermore, the complexity of the multielectron routes normally results in these processes requiring large overpotentials (i.e., high electricity consumptions), thereby largely limiting industrial applications. Second, the high stability of the CO_2_ molecule, with robust C=O bonds, normally results in sluggish kinetics and current density values well below those required for industrial applications. Despite the large variety of materials tested in this reaction including noble metals (e.g., Au, Pt, Ag, Pd), transition metals (e.g., Ni, Zn, Fe, and Cu), p-block metals (e.g., Pb, Sn, In, Bi), and doped carbon materials, the activity of the electroreduction reaction still remains unsatisfactory for practical applications [8,9]. Third, the CO_2_RR normally competes with an undesired and kinetically favored reduction process denoted as hydrogen evolution reaction (HER). The HER consumes electrical energy and protons in a secondary reduction reaction which normally takes place at potentials very close to (or even overlapping) those of some CO_2_RR products detailed above. Thus, the HER needs to be suppressed in order to improve the performance of the main CO_2_RR process [5,10].

Having these issues in mind, it is therefore crucial to develop new CO_2_RR catalysts combining: (i) high activity to convert the stable CO_2_ molecule, (ii) high selectivity (i.e., FE) towards a single product (preferably multielectron products), and (iii) the ability to suppress the undesired HER. In this sense, a new group of materials have recently emerged as an alternative to classical nanoparticle-based metal catalysts, the so-called single-atom catalysts (SACs) [11,12,13]. SACs consist of single metal atoms coordinated to a number of non-metal atoms, which provide anchor sites and provide stability to the otherwise unstable isolated metal atom. Among others, heteroatom-doped carbon materials, particularly those doped with N, are well suited for preparing SACs since N atoms incorporated into the graphene lattice are able to coordinate and stabilize single metal atoms [14,15]. This arrangement of single metal atoms is particularly convenient for the CO_2_RR process. First, it facilitates the exposure and use of the totality of metal atoms of the catalyst, providing very high activities and reducing overpotentials, which is highly convenient when targeting multielectron products [16,17]. Second, single-metal atoms present a unique electronic structure which, remarkably, can be finely tuned by changing their coordination environment. In particular, when stabilized in nitrogen-doped carbon materials, these single-metal atoms are reported to undergo deep changes in their electronic structure, with metal d orbitals being hybridized to a significant extent with the p orbital of carbon or nitrogen to stabilize its structure [18]. As a result, the adsorption energy of CO_2_RR reactants and intermediates over the single-metal atoms can be regulated with the coordination environment to drive the electroreduction process towards a desired product (i.e., selectivity improvement) [19]. Third, the special arrangement of single-metal atoms helps hinder the HER by suppressing the Volmer–Tafel route. This route requires two adjacent metal atoms, which is, by definition, very unlikely in SACs, thus forcing the reaction through the less favorable and higher activation energy Volmer–Heyrovsky route. As a result, unlike their nanoparticle-based metal counterparts, most SACs have been reported to favor CO_2_RR versus HER route [20,21]. Recent comprehensive overviews of electrochemical CO_2_ reduction and of single-atom catalysts in energy electrocatalysis further highlight the rapid development of this field [22,23].

Despite the significant advance that the use of SACs has entailed in addressing the limitations of CO_2_RR, it is necessary to move from a typical empirical trial and error-based catalysts screening towards a more mechanistically informed approach. In this context, the present review is aimed to shed light on by discussing the mechanisms by which the performance of SACs can be tuned through control of metal–support interactions, coordination environments, and rational design principles. As will be detailed in the following sections, metal–support interactions play a critical role in shaping the electronic properties of the metal atoms in SACs. The distinct reactivity of SACs in CO_2_RR arises from the pronounced sensitivity of the electronic structure of isolated metal atoms to the local coordination environment. Unlike nanoparticle-based catalysts, where metallic bonding among adjacent atoms results in delocalized electronic states and averaged reactivity, SACs present isolated metal centers whose d-orbital configuration is strongly influenced by the identity, number, and geometry of the surrounding ligands. These site-specific interactions can significantly modify the energies and occupancy of the metal d-orbitals and their alignment relative to the Fermi level, thereby altering the adsorption energies of key CO_2_RR intermediates on the metal site. As a result, even minor variations in support heteroatom doping or symmetry can induce substantial changes in catalytic behavior. The electronic structure of metal nanoparticles, on the other hand, is relatively robust against such perturbations owing to the collective nature of their electronic states [24]. This fundamental difference makes SACs especially amenable to rational design, enabling precise tuning of CO_2_RR activity and selectivity by modulating the affinity of the metal site for important CO_2_RR intermediates (e.g., *COOH, *CO, and *CHO) [18,25,26]. Interestingly, as will be analyzed in subsequent sections, the local metal coordination geometry (i.e., planar, pyramidal, or distorted) can be used to direct CO_2_RR towards a particular pathway, thereby allowing promote the formation of C_1_ vs. C_2_ products.

In this review, we aim to evaluate how these structural and electronic effects can be interpreted using selectivity descriptors (i.e., quantifiable parameters that correlate local atomic structure with CO_2_RR electrocatalytic performance). These descriptors can include coordination number, binding energy of key intermediates, oxidation state of the metal center, and hybridization between metal d orbitals and p orbitals of the support. In this sense, parameters such as the orbital-resolved electronic descriptors, such as the average energy of occupied d-states, and their positioning relative to the Fermi level, orbital overlap, and charge transfer characteristics can be used to predict the main CO_2_RR product (e.g., CO, formate, methanol, or more complex multicarbon products). The utility of these descriptors lies in their ability to simplify the complex multi-step reaction into a set of energetically dominant interactions, thus facilitating rational screening of catalyst candidates.

The present minireview therefore focuses primarily on N-doped carbon supports, examining how their porosity, heteroatom doping, curvature and local microenvironment modulate the coordination and electronic structure of isolated metal centers and, in turn, the adsorption and transformation of key CO_2_RR intermediates. Representative case studies on oxide and MXene supports are included only as selected examples to illustrate general principles of metal–support interactions that are transferable beyond carbon, rather than as an exhaustive survey of these families In selecting the literature, we prioritized studies that (i) explicitly connect structural or electronic features of metal–support interactions to CO_2_RR activity and selectivity, (ii) report quantitative metrics under relatively practical conditions (e.g., FE and current density), or (iii) introduce generalizable concepts such as curvature engineering, hydrophobic gas-diffusion architectures, or electronic metal–support interactions. The emphasis was put on extracting design principles and highlighting remaining gaps.

## 2. Mechanistic Pathways and Product Selectivity in CO_2_RR

**Figure 1:**
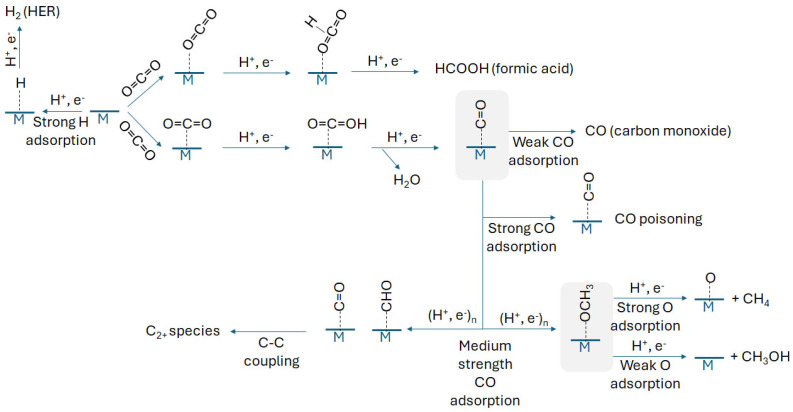
Reaction pathways and intermediates involved in the production of C_1_ and C_2+_ products by CO_2_RR on SACs. The adsorption strength of key intermediates such as *COOH, *CO, and *CHO on single metal atoms determines the course of the reaction. M denotes the single metal atom center of the SAC (e.g., Fe, Co, Ni, Cu).

CO_2_RR is a complex process involving a sequential series of electron and proton transfers which can result in a diverse array of chemical products. These products broadly fall into two categories namely, C_1_ (e.g., carbon monoxide, formic acid, methane, and methanol) and C_2_ (e.g., ethylene and ethanol) compounds. The specific reaction route followed and, thus, the resulting mixture of products is primarily determined by the nature of the chemical species adsorbed onto the catalyst surface and how strongly these intermediates are stabilized under the reaction conditions. In the context of SACs, CO_2_RR typically starts with the adsorption and subsequent activation of the CO_2_ molecule (Figure 1). First, CO_2_ can be adsorbed on the metal through oxygen (*OCO/HCOOH-like) or carbon (*CO_2_) atoms depending on the nature of the metal (M in Figure 1). For oxophilic metals (e.g., Bi, Sn, In), DFT and operando studies support the formation of O-bound HCOO-like intermediates leading to formate, rather than a linear M–OCO species [27,28]. Adsorption via *CO_2_ leads the reaction to the formation of a *CO intermediate after reduction and water removal steps. Once CO is formed, it can either detach from the catalyst surface as a stable C_1_ product (when it is weakly adsorbed on the metal), or it can undergo further reduction provided that the adsorption over the metal is sufficiently high. This further reduction of *CO can lead to more hydrogenated C_1_ compounds (e.g., methanol or methane) through the formation of additional adsorbed intermediates such as *CHO, *CH_2_O, and *CH_3_O. Conversely, the generation of C_2_ products such as ethylene and ethanol requires a C–C bond formation step. As shown in Figure 1, for this step to take place is generally necessary the accumulation of *CO or *CHO intermediates on adjacent or closely situated active sites. Although SACs are, by definition, isolated metal atoms and thus lack intrinsically adjacent metal sites, C–C bond formation can still proceed, as we will describe in following sections. This is facilitated through surface-mediated coupling mechanisms, particularly when the active site environments are confined or when dual-metal motifs are present within the catalyst structure [1,26].

As can be inferred from the above discussion, the strength of adsorption of certain key CO_2_RR intermediates on metals can provide relevant information on the preferred pathway through which the reaction will take place. In this sense, the binding energies of CO_2_RR intermediates on transition metal catalysts have been reported to be strongly correlated with each other in virtue of the so-called “scaling relationship” [29]. According to this, the adsorption energy of one particular intermediate (e.g., *CO) can be selected as a representative for linear scaling correlations with the other intermediates of the reaction. Thus, the adsorption strength of CO on the metal catalyst can be used as an excellent indicator for predicting the course of the reaction [30].

Theoretically, metals adsorbing CO weakly are likely to release CO once the *CO intermediate is formed, generating CO as the main product, whereas metals showing strong CO adsorption are likely remaining on the metal surface undergoing subsequent electroreduction processes or even blocking the metal surface by CO poisoning [31,32] (Figure 1). CO blocking should therefore be always taken into consideration when running CO_2_RR, considering the low temperatures at which the reaction is carried out. Thus, only metals with medium CO adsorption strengths will allow *CO to be reduced to a *CHO intermediate and then to *OCH_3_ after several reduction processes [33]. For example, Cu, characterized by a moderate affinity for CO, is particularly well suited to stabilize *CO without overbinding, thereby enabling its subsequent hydrogenation to *CHO and the subsequent production of multielectron C_1_ products such as methane or methanol [34]. As shown in Figure 1, the *OCH_3_ intermediate still can undergo further reduction, with metals showing strong oxygen adsorption converting this *OCH_3_ intermediate into methane, leaving the metal atom blocked by strongly chemisorbed *O species. On the other hand, metals combining medium CO adsorption strengths and weak oxygen adsorption are likely to allow complete desorption of the *OCH_3_ intermediate upon reduction, generating methanol and leaving the metal site free for subsequent reaction cycles. Finally, as shown in Figure 1, metals with high tendency to adsorb hydrogen are to be avoided since they will end up promoting the undesired HER. Only those metals with weak (or none) hydrogen adsorption should therefore be considered to ensure that competing reduction processes are not promoted over the metal sites [35].

As stressed in the Introduction, given the isolated nature of the metal site, the interaction of SACs with CO_2_RR intermediates is very specific and highly adjustable. Thus, the thermodynamic stability of the intermediates and thus the final product distribution is not solely controlled by the metal atom but also profoundly influenced by the specific coordination geometry of the metal and its electronic structure, both of which are largely determined by the supporting material [25]. These factors play a decisive role in stabilizing or destabilizing various reaction intermediates, thereby selectively promoting or inhibiting certain reaction pathways. Thus, the formation of *COOH or *HCOO intermediates and therefore the course of the CO_2_RR reaction will depend on specific electronic interactions between the metal atoms and the activated CO_2_ molecule. The *HCOO intermediate, which leads to formic acid, involves σ-type interaction between the metal and the oxygen atoms, and is typically stabilized on oxophilic or p-block metal sites. In contrast, the *COOH intermediate, which evolves into *CO and potentially into CO, methane, methanol and C_2_ products, is stabilized on those metals or metal–nitrogen coordination environments favoring π-backbonding with the carbon center. Among this route, the reduction of *CO into *CHO is generally considered the first thermodynamically uphill step in the C_1_ hydrogenation pathway and often represents the rate-determining step when targeting products such as methanol or methane [36]. The stabilization of this *CHO intermediate is highly sensitive to the electronic characteristics of the active site, particularly in single-atom configurations where ligand field effects can shift the binding energy landscape dramatically. Thus, the coordination environment of the metal (e.g., M–N_4_, M–N_3_C, or M–N_2_S) not only stabilizes the otherwise isolated metal atom but also alters its d-electron distribution and Fermi level alignment [37,38]. These electronic perturbations significantly impact the adsorption energies of key intermediates such as *COOH, *CO, and *CHO. For instance, altering the planar Cu-N_4_ coordination of a metal center by boron doping can tune its electronic structure to enhance the binding of key intermediates *CO, and *CHO, thereby promoting efficient CO_2_ reduction to CH_4_ [39].

The formation of C_2_ products requires additional molecular-level complexity. Thus, apart from the accumulation of surface-bound *CO species, successful C–C coupling relies on the co-adsorption of intermediate pairs such as *CO–*CO or *CO–*CHO on adjacent sites [40]. These combinations facilitate coupling via intermediates such as *OCCO or *CHCO, eventually giving rise to *CH_2_CHO or *CH_3_CH_2_O species, which can undergo further reduction to yield ethylene or ethanol. These C_2_ routes involve transition states that are strongly affected by the geometric constraints of the catalytic sites [41]. Apart from the geometrical limitations, the formation of C_2_ products typically requires multiple sequential electron-proton transfer steps, which significantly increases the kinetic barrier of the overall process. Consequently, these transformations generally occur at higher overpotentials compared to C_1_ product formation, resulting in increased energy input and lower FE values [42,43]. In the case of SACs, the discrete nature of the active site often imposes strict geometrical constraints, making the identification and characterization of adsorbed species especially critical for rational catalyst design [44].

## 3. Physicochemical Characteristics of SAC Supports and Their Influence on the CO_2_RR Performance

The physicochemical characteristics of the support material have a large influence on the behavior of SACs in the CO_2_RR. In this sense, textural features of the carrier such as surface area, porosity, and capacity for mass transport have been shown to greatly alter the performance of single atoms, particularly those anchored on carbon supports. Recent studies have revealed that optimization of these factors is equally important as manipulating electronic or chemical microenvironments at the atomic sites, especially to reach industrially acceptable current densities and selectivities [45]. Hierarchically porous carbon supports, containing both mesopores and micropores, significantly enhanced the accessibility of single atomic sites, allowing rapid CO_2_ diffusion to active centers, whereas supports containing only microporous or being non-porous showed poorer diffusion rates for both CO_2_ and electrolytes, an issue that becomes important at high current densities [9,45,46]. This improvement in CO_2_ diffusion is reflected in enhanced electrocatalytic performance. For example, Li et al. [47] prepared a Ni-NOMC-1 catalyst showing interconnected micropores (ca. 0.6 nm), mesopores (ca. 50 nm), and macropores (ca. 200 nm) that achieved nearly 100% FE towards CO over a wide voltage window (0.2 to 1.1 V vs. RHE) in flow cells, delivering industrially relevant CO partial current densities (208 mA cm^−2^). This catalyst outperformed its microporous-only analogs, which delivered significantly lower current densities, TOF, and selectivity under the same conditions. Shao et al. [9] used PVP-modulated MOF-derived carbonization to prepare hierarchical Ni-N-CP-8 SACs with double TOF towards CO than their corresponding microporous counterparts. Electrochemical impedance, product analysis, and molecular dynamics simulations confirmed the importance of having hierarchical pore structures to low transport resistance. Despite their high selectivity, the CO partial current densities of these Ni–N–C SACs remain below the levels typically targeted for device-relevant operation, suggesting that further optimization of catalyst-layer architecture and mass transport is required to fully exploit their intrinsic activity. Wen et al. [45] used rapid microwave-assisted MOF pyrolysis to prepare mesoporous/defective Ni_1_NC-50 SACs, which delivers an outstanding CO current density of 1.06 A cm^−2^ with a FE of 96% in flow cell operation. Microporous counterparts with similar active site loadings operating under identical conditions showed very poor CO density currents of 0.23 A cm^−2^ at 93% FE, thereby highlighting the importance of meso/macroporous textural control on the carbon support for high-rate industrial applications. This trend was confirmed by Zhao et al. [48] over Cu SACs prepared by ZIF-8 MOF carbonization in the production of C_3_ condensation products (e.g., acetone). DFT and spectroscopy confirmed that both the carbon porosity and the local N coordination environment of Cu played a significant role in the selective pathway towards acetone. The importance of porous carbon supports (carbon nanotubes, “multi-channel” carbon matrix, carbon paper, etc.) in forming effective interfaces for transition metal SACs (e.g., Co, Sn, Cu, and Bi-based) electroreduction catalysts was also highlighted by Ai et al. [49]. Only the catalysts synthesized on highly porous supports achieved high CO_2_RR current densities.

**Figure 2:**
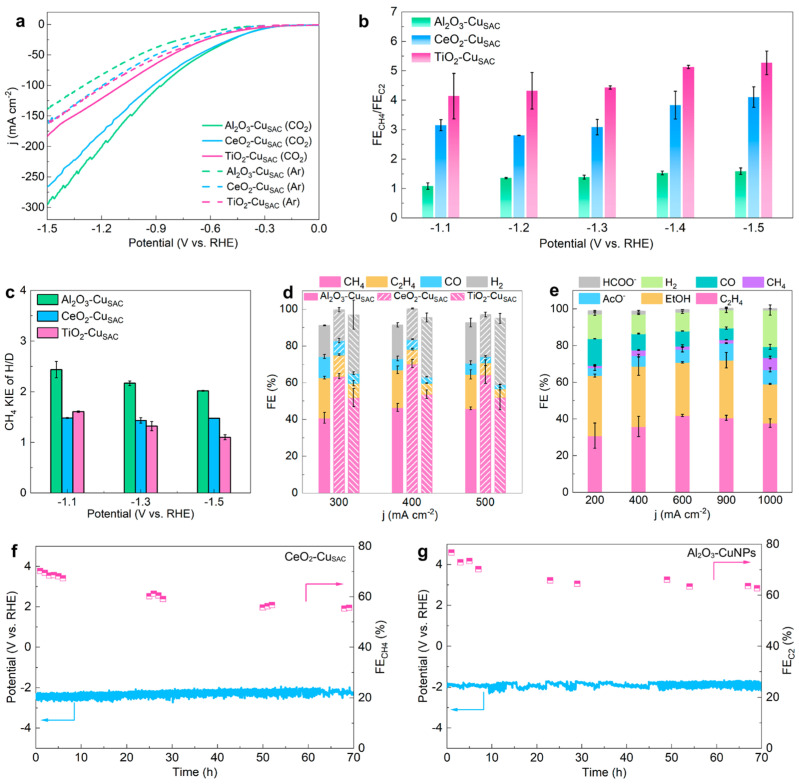
CO_2_RR performance of Cu SACs supported on Al_2_O_3_, CeO_2_ and TiO_2_. (**a**) Polarization curves for Al_2_O_3_-, CeO_2_- and TiO_2_-supported Cu SACs in CO_2_-saturated and Ar-saturated electrolytes. (**b**) Ratio of CH_4_ to C_2_H_4_ + C_2_H_6_ FEs as a function of potential, highlighting the different C_1_/C_2_ selectivities induced by the oxide support. (**c**) Kinetic isotope effect (KIE) for CH_4_ formation, evidencing distinct rate-determining steps on the three SACs. (**d**,**e**) Product distribution (FE) versus current density, comparing Al_2_O_3_-, CeO_2_- and TiO_2_-supported Cu SACs with Al_2_O_3_-supported Cu nanoparticles, and illustrating the transition from H_2_-dominated to C_2_-rich products. (**f**,**g**) Long-term electrolysis tests showing the stability of CeO_2_-Cu SACs and Al_2_O_3_-Cu nanoparticles under high-current operation. Reproduced from [6] under the terms of the Creative Commons Attribution 4.0 International License (CC BY 4.0).

Apart from the textural properties, controlling the electronic properties at the interface of metal and support plays a crucial role in controlling the activity, selectivity, and product distribution of SACs for CO_2_RR. In particular, charge transfer and electronic metal–support interaction (EMSI) can alter the density of states near the Fermi level, finely tuning the adsorption strengths of intermediates over the atomic site and the extent of electron delocalization that determines product selectivity and suppression of parasitic reactions. In an excellent work, Zhang et al. [6] demonstrated, by rigorous experimental and DFT studies, that the nature of the oxide support (e.g., Al_2_O_3_, CeO_2_, TiO_2_) altered charge transfer dynamics towards the Cu metal, leading to different Cu d-orbital configurations and thus different DOS near the Fermi level, which ultimately defined product distribution. As shown in Figure 2a, all three CuSACs delivered high current densities in CO_2_-saturated electrolytes, evidencing strong CO_2_RR activity. Quantitative product analysis (Figure 2b–e) revealed that Al_2_O_3_-CuSAC favored C–C coupling, leading particularly to C_2_H_4_ and C_2_H_6_ as the current density increased. Reducible oxides such as CeO_2_ and TiO_2_ guided the reaction towards different pathways. Thus, CeO_2_-CuSAC stabilized *COOH and related intermediates, thereby resulting in high FE toward methane, while TiO_2_-CuSAC predominantly promoted H_2_O activation and H_2_ formation via HER. CH_4_ kinetic isotope effect data (Figure 2c) further revealed the different rate-limiting steps for methane production on the three supports, in line with the different electronic structures revealed from DFT. Finally, long-term electrolysis tests (Figure 2f,g) revealed that CeO_2_-CuSAC produced methane with high selectivity and stable potential over tens of hours at industrially relevant current densities (up to 400–500 mA cm^−2^), whereas Al_2_O_3_-supported Cu nanoparticles showed different stability and selectivity profiles.

SACs anchored on polyoxometalate (POM) supports confirmed the importance of metal transfer dynamics on the CO_2_RR performance of metal SACs [50]. Systematic DFT analysis revealed electron transfers from the active metal site to adjacent O atoms in the POM, which resulted in altered DOS near the Fermi level and enhanced binding and activation of reaction intermediates. In particular, PDOS calculations for Cr-based SACs revealed strong d–p orbital mixing near the Fermi level, which resulted in remarkable reactivity and selectivity towards formic acid. Charge redistribution between the metal and support was found to promote both CO_2_ activation and product selectivity. In the same line, Jin et al. [51] studied 3d single metal atoms (e.g., Sc, Ti) supported on graphene vacancies and analyzed the charge transfer and projected density of states (PDOS) near the Fermi level. Significant overlaps between metal d-states and graphene π states were found. In addition, the position of the energies and occupancy of the metal d-orbitals relative to the Fermi level was found to strongly impact the strength of intermediate adsorption, HER inhibition, and product selectivity. A detailed Bader charge analysis and PDOS maps directly correlated the electronic structure tuning with the observed CO_2_RR activity and selectivity towards HCOOH.

Chemical modifications of the support such as heteroatom doping (N, S, P, B), introduction of vacancies and tuning of surface functional groups (–OH, –NH_2_) have been actively explored to enhance the catalytic activity and selectivity SACs for CO_2_RR. In this sense, doping with heteroatoms or creating vacancies can shift local charge density and modulate the orbital distribution of active sites, thereby promoting more favorable adsorption and activation of CO_2_ and intermediates. Heteroatom doping of the support has been shown to be particularly effective in tuning the electronic environment of the metal site. Yue et al. [3] explored P-doping around a SnN_4_–CNT system and found that the electronic structure of Sn was significantly altered, promoting the reduction of Sn^4+^ to Sn^2+^ and improving CO_2_ activation, as confirmed by both in situ and ex situ spectroscopic measurements. The P-doping resulted in a SAC with outstanding CO partial current density (ca. 380 mA cm^−2^) and FE (>98%) values. Si-doping nearby Fe-N_4_ sites on carbon was found to be crucial in mitigating CO poisoning over Fe, resulting in industrially relevant current densities (350 mA/cm^2^ and 100% FE towards CO [52]. Si-doping resulted in a negative shift in the Fe d-band and a weaker CO adsorption, as supported by DFT and x-ray adsorption spectroscopy (XAS). Porous carbon was doped with both N and O, allowing NO coordination around single K atoms [53]. As a result, hybridization of the K sp-orbitals with O/N p-orbitals was induced, substantially altering the charge state of the K sites, which otherwise were catalytically inactive. Outstanding FE values of 97.8% towards CO and superior stability were found after doping. The effects of B, N, S, and O heteroatom doping and controlled vacancy generation over SACs were methodically studied [50]. Regardless of the metal, this DFT study showed that charge redistribution (analyzed via Bader charge and partial DOS), induced by dopants or vacancies, tunes the electronic structure of atomic metal sites, enabling them to optimally bind, activate, and hydrogenate CO_2_, especially when surface groups (–OH, –NH_2_) are present near the atomic sites.

Introduction of vacancies on the carbon support represents an interesting approach to significantly change the interaction of CO_2_ with atomic metal sites. As demonstrated by Yang et al. [54], the introduction of vacancy sites on a carbon nitride support (g-C_3_N_4_) via exfoliation and thermal treatment, resulted in stable Nd-N_6_ coordination sites with high FE towards CO and outstanding stability. DFT calculations revealed that vacancies induced strong anchoring of Nd atoms, leading to enhanced electron transfer, *COOH intermediate stabilization, and high stability with no signs of metal agglomeration after 50 h of electrolysis. Abundant N and C vacancies were intentionally created on a ZIF-8 based carbon support by a high-temperature treatment [55]. The vacancies served as ideal anchoring points for Ni atoms, allowing high atomic dispersion and Ni-N_3_ coordination. The catalyst achieved outstanding FE values to CO (98.2%) and excellent long-term electrochemical and structural stability. Raman and BET analyses confirmed the critical role of vacancy-rich frameworks in structural disorder, high surface area, and catalytic performance. However, despite the excellent FE and stability, the reported CO partial current densities remain below device-relevant values, indicating that mass-transport and catalyst-layer engineering still limit the practical exploitation of these Ni–N–C single sites.

**Figure 3:**
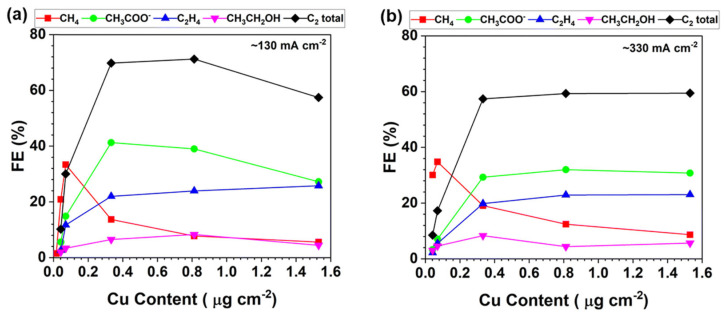
Evolution of product selectivity for the CO/CO_2_ reduction reaction on hydrophobic carbon gas diffusion electrodes as a function of atomically dispersed Cu loading. Increasing Cu loading on the hydrophobic GDE shifted the selectivity from CH_4_ to C_2_-rich products (CH_3_COO^−^, C_2_H_4_, CH_3_CH_2_OH) with total C_2_ FEs up to: (**a**) ca. 78% at -130 mA/cm^2^ and (**b**) ca. 60% at -330 mA/cm^2^, while hydrogen evolution remains suppressed. Reproduced from [57] under the Creative Commons Attribution–NonCommercial 3.0 Unported license (CC BY-NC 3.0).

Manipulation of the interfacial environment by controlling the hydrophobicity, hydrophilicity, and wettability has also been used to direct the complex CO_2_RR towards desired products. In particular, by tailoring the way water interacts with the atomic site surroundings, it is possible to inhibit the undesired HER while enhancing local CO_2_ concentration at the active sites. Perhaps one of the most relevant works using this approach was recently published by Zhang et al. [7]. Hydrophobic CuCu_2_O/Ag-SAC catalysts were prepared by a careful control of surface chemistry during the electrodeposition process. The hydrophobicity of the surface resulted in superior CO_2_ adsorption and hindered water access, thereby favoring CO_2_RR over competing HER. The resulting catalyst delivered high FE to C_2_^+^ products. Systematic contact angle measurements, DFT calculations, and CO_2_RR testing revealed a direct link between the hydrophobicity of the catalyst surroundings and the performance of the atomic sites in this reaction. This approach has been commonly used to promote C-C coupling reactions while suppressing HER. Chala et al. [56] came up with a strategy to manipulate the wettability of nitrogen-doped carbon supports, which facilitated local CO enrichment at Cu single atom sites. CO retention, as indicated in Section 2, is required to direct the synthesis to valuable methanol or C-C coupling products. As a result, C-C coupling to ethanol (FE as high as 71%) was promoted over tandem Co-Cu atomic sites and HER was effectively suppressed, as revealed by the very low H_2_ productions obtained. In line with these results, Lyu et al. [57] demonstrated that selectivity control and HER suppression can be regulated by surface microenvironment engineering and wettability tuning. The hydrophobic character and wettability of carbon gas diffusion electrodes was found to play a direct role in tuning product distributions and limiting HER. The atomically dispersed Cu sites on hydrophobic carbon gas diffusion electrodes showed high selectivity to C_2_ products (up to 78% Faradaic efficiency) while suppressing HER. As shown in Figure 3, both high hydrophobicity and high atomic Cu loadings were found to be essential for maintaining high selectivities to condensation products and current densities. Thus, at very low Cu loadings the CO electroreduction on hydrophobic carbon produced predominantly CH_4_, while higher atomically dispersed Cu loadings resulted in a gradual transition toward C_2_ products (CH_3_COO^−^, C_2_H_4_, and CH_3_CH_2_OH). This behavior highlights that the hydrophobic electrodes not only suppressed HER but also enables efficient utilization of trace Cu sites, provided that the atomic loading is high enough to sustain dense local *CO coverage and facilitate C–C coupling. The exceptionally high activities and TOFs reported for both CH_4_ and C_2_ formation emphasized how microenvironment wettability and controlled Cu dispersion can be used to improve the intrinsic performance of Cu-based SACs well beyond that of conventional nanoparticle catalysts.

Similar approaches modifying surface polarity and oxophilicity to direct the CO_2_RR process have also been highlighted in recent works [58,59]. Deliberate introduction of local oxophilic or hydrophobic domains on catalyst surfaces generated preferential CO_2_ adsorption and facilitates water exclusion at the reaction interface. In situ and operando spectroscopies and electronic structure calculations, revealed that hydrophobic domains helped segregate water, while oxophilic “islands” guided the adsorption pathway and electron transfer, enhancing C–C coupling and suppressing HER.

The solvation structure of cations and additives in the electrolyte can also be used to regulate the microenvironment around the catalytic sites. Recent studies by Ni et al. [60] and Wang et al. [61] showed that careful control of the solvation structure of cations and additives can be used to regulate the strength of water-cation interactions, which ultimately led to suppression of undesired proton transfer steps (and thus HER inhibition) and a promotion of key CO_2_RR intermediates, as confirmed by in situ spectroscopies (ATR-IR, Raman) and DFT calculations. These studies illustrate that tailoring the electrolyte microenvironment can be used to boost the activity and selectivity of SACs in the CO_2_RR, although its practical implementation will require robust, scalable formulations that maintain performance under high current-density operation.

**Figure 4:**
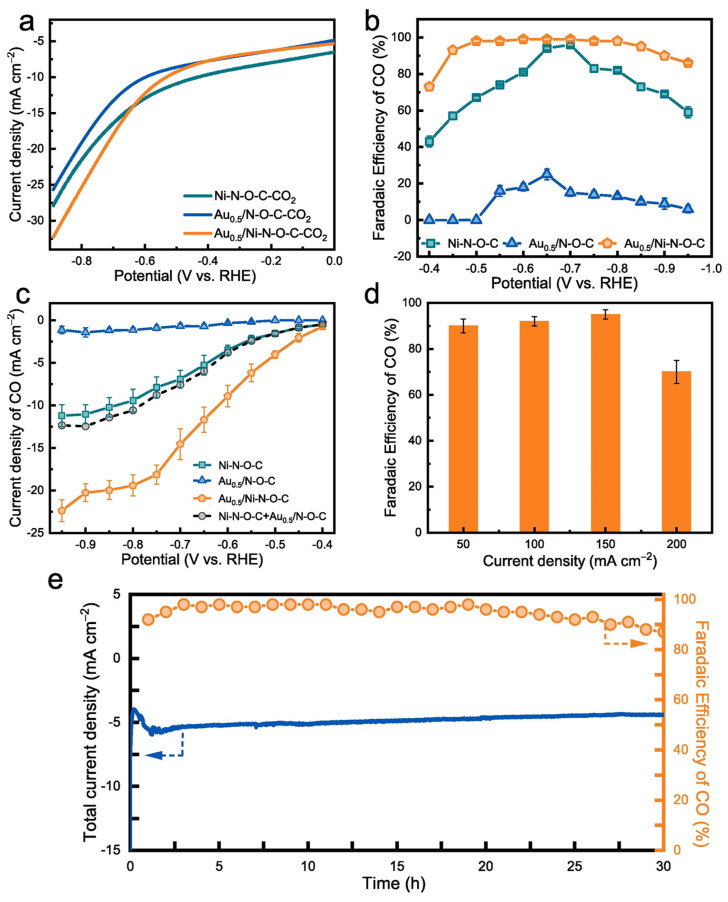
CO_2_-to-CO electroreduction performance of Ni–N–O–C single-atom catalysts and Au-modified Ni–N–O–C configurations. (**a**) Polarization curves in CO_2_-saturated electrolyte (**b**) CO FEs versus potential, where Au_0_._5_/Ni–N–O–C reaches near-unity CO selectivity across a broad potential window. (**c**) CO partial current density as a function of potential, highlighting the synergistic enhancement from mixed ligand (N, O, C) coordination and neighboring Au species. (**d**) CO FE at different current densities, showing high selectivity up to 200 mA cm^−2^. (**e**) Long-term stability tests, showing sustained CO selectivity and nearly constant total current density over prolonged electrolysis. Reproduced from [63] licensed under the Creative Commons Attribution 4.0 International License (CC BY 4.0).

The operational stability of SACs is crucial for their practical application in the CO_2_RR. However, the isolated nature of their active sites makes them particularly susceptible to degradation. Thus, the harsh electrochemical environment including redox cycling and the presence of aggressive ionic species can result in rapid metal aggregation (sintering) and leaching from the support into the electrolyte. The support material can be engineered to mitigate metal sintering/restructuring and leaching issues. Thus, the local coordination environment in the support (e.g., number and identity of atoms around the metal center) has been found to directly affect the binding energy between the metal and support, which in turn governs the resistance to atom migration, agglomeration, or leaching. [62]. For example, when N-doped graphene materials are used as supports, pyridinic and porphyrin-like ligands having multiple N or mixed N and C atoms linked to the metal resulted in high metal-support binding energies, robustly anchoring the metal atom and thereby suppressing mobility. Supports like graphitic carbon nitride (g-C_3_N_4_) provide abundant pyridine-like nitrogen sites that localize and stabilize rare earth single atoms through N_6_-coordination cages. This coordination architecture was found to be particularly effective in preventing metal agglomeration under high current conditions [54]. Stabilization via mixed (N and C or O) coordination with the metal were found to be optimal since it combines electronic stabilization and geometrical accommodation [63]. This concept is shown in Figure 4, where the Ni–N–O–C coordination environment, further modulated by adjacent Au species, delivered both higher current densities and CO FEs over a wide potential and current-density range compared with the parent Ni–N–O–C site. The mixed coordination not only enhanced activity but also sustains high CO selectivity and stable operation over extended electrolysis times, highlighting how properly engineered heterogeneous ligands can stabilize SACs under CO_2_RR conditions.

Intentional generation of vacancies, heteroatom doping, and structural disorder to the carbon support have also been exploited to maximize both the number of stable SAC sites and their resistance to agglomeration and electrochemical deactivation during operation [64]. Thus, the creation of porous, defect-rich supports with multiple anchoring sites (e.g., vacancies, dopants) was found to increase energy barrier for metal migration. The presence of large vacancy sites (e.g., multi-vacancy or N-rich edges) was found to increase the 3d transition metal-atom stability, as revealed by DFT [51]. Apart from carbon supports, theoretical calculations have revealed polyoxometalates (POMs) such as SiMo_12_O_40_ as excellent anchoring materials for SACs [50]. Early transition metals (e.g., Sc, Ti, V, Cr, Mn) were found to form negative adsorption energy bonds with the POM 4-H site, which were confirmed to be stable at elevated temperatures by ab initio molecular dynamics (AIMD) simulations. Electrochemical Pourbaix diagrams further demonstrated that these SACs resist OH^−^ coverage and oxidation/leaching under CO_2_RR conditions, as long as the electrode potential is below certain values. Taken together, these results indicate that POM anchoring can, in principle, enable electrochemically robust early-transition-metal SACs, although the narrow potential window for stability may limit their direct translation to high-current, industrially relevant operation.

## 4. Rational Design of SACs: Metal–Support Interactions

The rational design of SACs for CO_2_RR is based on the modification of the electronic environment of the atomically dispersed active site by both the nature of the support and the strength and type of metal-support interactions. In this section we will discuss how, through deliberate medication of the support (e.g., carbon materials, metal oxides, MXenes, nitrides, MOFs, etc.), researchers can finely tune electron density and metal-support orbital overlap, thereby improving the electrocatalytic performance and directing the synthesis towards desired valuable products. As will be detailed in this section, manipulating metal-support interactions in SACs can be achieved by a number of approaches including dopant selection (e.g., N, S, P, B), defect and vacancy engineering, and the creation of mixed-ligand and tailored coordination environments (M–N_4_, M–O_4_, M–S_4_, etc.), which enables precise control over the electronic structure and reactivity of the active site. This section systematically reviews the most significant advances, categorizing strategies by support class and illustrating how fine-tuning the support can help obtain the full potential of SACs in this reaction.

### 4.1. Carbon-Based Supports

Carbon-based supports are, by far, the most frequently used for SACs owing to their excellent conductivity, high surface area, chemical tunability, and wide variety of coordination geometries. Insertion of nitrogen and other heteroatoms (e.g., S, P, B) into the carbon frameworks allows stabilizing isolated metal atoms while tuning their electronic environment. A wide variety of carbon materials including graphene [62,65,66], carbon nanotubes (CNTs) [3], carbon nanofibers (CNFs) [67], hollow nanocubes [54,55], carbon spheres [68,69], and metal–organic framework (MOF or ZIF)-derived carbons [55] have been used for this purpose. The interactions between the atomic metal sites and the carbon support are mostly determined by the first coordination shell surrounding the metal. DFT studies have consistently demonstrated that the first coordination shell is the dominant factor controlling both the metal binding energy and its electronic state [62]. Thus, the specific coordination of graphitic N, pyridinic N, and/or C ligand in the first shell can lead to significant changes on the adsorption energy for CO_2_ and key reaction intermediates (e.g., *COOH and *OCHO), with a clear impact on product selectivity. For example, in the case of Fe, Ni, and Cu SACs, the coordination with pyridinic N resulted in higher adsorption energies for the reaction intermediate *COOH, thereby lowering the activation barrier for the electroreduction of this intermediate (considered the rate determining step of the CO_2_RR). As a result, higher turnover frequencies were obtained while lowering the onset potential for CO formation. On the other hand, when graphitic N was replaced by C in the first coordination shell of the metal site, the adsorption profile of CO_2_ and intermediates changed dramatically, with the *OCHO intermediate being adsorbed with optimal binding energies and selectivity shifted from CO to HCOOH as a result. These DFT predictions have been later confirmed by experimental works [3,55,63], thereby revealing the importance of adding N and other heteroatoms (e.g., O, P) to the coordination sphere of the metal to lead the CO_2_RR through the *COOH intermediate. Insertion of Si in the second coordination sphere of Fe-N-C sites significantly modulated the electronic structure of the metal, resulting in weaker CO adsorption and its release via formation of *COOH intermediate, as revealed by in situ ATR [52]. In line with DFT predictions, FE towards CO close to 100% were obtained at −0.4 V, with density current values as high as 350 mA/cm^2^.

Apart from the coordination sphere, the geometrical shape and local curvature of the carbon support (e.g., planar graphene versus cylindrical CNTs) have been found to have a significant effect on the CO_2_RR performance of SACs. For example, Zhang et al. [70] studied the influence of the curvature of the carbon support on the electronic properties, coordination environment, and catalytic activity of Fe and Cu SACs.

**Figure 5:**
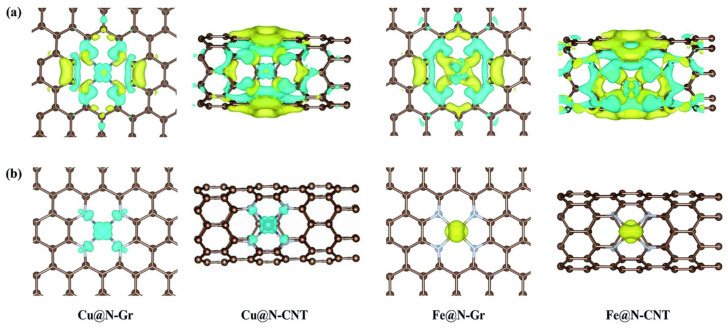
Curvature-induced modulation of charge distribution for atomically dispersed Cu and Fe on N-doped carbon supports. (**a**) Charge density difference isosurfaces for Cu@N-Gr and Cu@N-CNT, highlighting how the transition from a flat graphene lattice to a curved CNT alters electron redistribution between the Cu center and surrounding N-doped carbon. (**b**) Corresponding charge density difference isosurfaces for Fe@N-Gr and Fe@N-CNT, showing a distinct curvature response relative to Cu. Yellow and cyan regions denote charge accumulation and depletion, respectively. Reproduced from [70] licensed under the Creative Commons Attribution–NonCommercial 3.0 Unported License (CC BY-NC 3.0).

As shown in Figure 5, the curvature of the support altered the overlap between the d orbitals of the central metal atom and the p orbitals of the surrounding N or C atoms, resulting in significant changes in the electronic density around the metal site. These factors crucially affected the stabilization of key intermediates such as *COOH and *OCHO during the reaction. Thus, DFT revealed that, for a similar FeN_4_ site, the adsorption energies for CO_2_ and critical intermediates were very different when considering graphene (flat) and CNTs (curved), leading to different limiting potentials (*COOH or *OCHO formation) and thus different selectivities for CO or formate, respectively. The flatness of graphene was found to provide a stable, planar coordination environment with specific N–M–N bond angles and orbital overlap optimal for *COOH activation and CO release, whereas the curvature of CNTs distorted and compressed the first coordination shell, altering both local electronic density and orbital orientation of the atomic metal site. This alteration was found to stabilize *OCHO intermediates more than *COOH, thereby directing the synthesis towards formate. Interestingly, the curvature effect was particularly pronounced for Cu as compared to Fe, with CNTs promoting a shift in selectivity from CO and to formate and methanol via stabilization of specific intermediates by the distorted geometry. This work provides an outstanding example on how the choice between flat or curved carbon supports can be used for steering catalytic selectivity in CO_2_RR. Wang et al. [71], on the other hand, found high-curvature carbon nanofibers to favor CO production over Zn–N_4_ sites via a strong overlapping between the carbon matrix and the Zn 3d orbitals, which resulted in higher electron density at the Zn site and stronger *COOH adsorption as compared to a control planar graphene material. These results were in line with those obtained by Wang et al. [72] over Co SACs. Curvature was also found to inhibit Co aggregation, thereby improving stability during operation. Curvature of the carbon support also favored the formation of higher hydrocarbons over V SACs [73]. Combined DFT and experimental results revealed the introduction of high curvature to a 2D BC_3_N_2_ support to substantially modify the local geometric coordination and electronic properties of V atomic sites. The high curvature resulted in electron redistribution, which lowered the energy barrier for C-C coupling and *COOH/*CHO formation. This enabled efficient and selective production of methane, ethylene and ethanol. Similar results were found by Chen et al. [72] over Ni and Fe SACs. Adjustment of the curvature of CNTs was used to modulate the energies and occupancy of the metal d-orbitals of the metal, with greater curvature increasing the overlap between the metal and the support orbitals, lowering the activation barriers for *COOH formation and C-C coupling. Highly curved CNT-supported catalysts outperformed their flat or low-curvature counterparts in both current density and C_2_^+^ hydrocarbon selectivity.

The textural properties of the carbon support (e.g., porosity, pore size distribution, and BET surface area) also have a strong influence on the electrochemical performance of SACs. Thus, supports with high BET surface area and hierarchical porosity were found to increase the density of exposed and accessible single-atom sites and promote mass transport of CO_2_ and protons. Also, fast desorption of gaseous products (e.g., CO) was allowed, mitigating product poisoning and boosting stability, resulting in superior FE towards CO and cell stability [55]. The presence of mesoporosity and high BET area in carbon nanofibers was found to improve current density via faster CO_2_ and electrolyte diffusion, further emphasizing the critical role of the support porosity in CO_2_RR [74].

Surface functionalization with oxygen groups (e.g., –OH, –COOH) have also been used as a strategy to improve the performance of SACs. Shao et al. [9] functionalized carbon supports with surface oxygen containing groups by varying the carbon precursor and the activation conditions. Functionalization of the carbon support resulted in surface -OH/-COOH functionalities improving electrolyte wetting and dispersion of Ni–N_x_ sites. The functionalized material provided significantly higher current densities at similar FE towards CO, which was ascribed to improved mass transport and surface wetting rather than changes in first-shell coordination. Han et al. [63] provided a very elegant approach to promote electron delocalization via N and O surface functionalization of the carbon support. Thus, the carbon support was co-doped with N and O generating as a result abundant pyridinic N, graphitic N, and C–O/C=O sites around the active Ni–N_3_O centers. These N/O groups integrated into the carbon framework were found to act as extended electron-delocalization channels rather than simply first-shell ligands. XANES/EXAFS and DFT revealed that neighboring O functionalities hybridized with Ni–N_3_ states, resulting in an “electron-tug” effect that redistributed electron density over the π-system. Remarkably, this electronic modulation lowered the energy barrier for *COOH formation while also weakening *CO adsorption to promote facile desorption of CO and suppression of HER. High FE towards CO (>95%) at high current densities with excellent long-term stability were found. This work is an excellent example that surface functionalization of the carbon support beyond the immediate first coordination shell can be used as a valid strategy for optimizing CO_2_RR activity and selectivity on Ni SACs. In a similar fashion, Qu et al. [53] used a porous carbon support rich in N and O functional groups to coordinate isolated K sites. The type and distribution of N/O sites was controlled by the carbon precursor and the thermal treatments. DFT and ATR-IR revealed that NO coordination around K dramatically lowered the barrier and overpotential for *COOH formation, a step highly unfavorable on pristine carbon or N-only functionalized carbons. The polar N/O functionalities also helped tune local pH and proton availability near K sites, thereby facilitating CO_2_ activation while suppressing HER. As a result, FE towards CO surpassed 90% over a wide potential range as compared to the unfunctionalized counterparts. Thus, tailored N/O surface chemistry was used to boost the intrinsically poor activity of a s-block metal single-atom catalyst.

### 4.2. Oxide Supports

Apart from carbon, a variety of oxide-based supports—including oxide clusters, extended oxides, and oxyhydroxides—have been explored for CO_2_RR on SACs. These materials are attractive because of their tunable reducibility, defect chemistry, and acid–base properties can modulate the oxidation state, orbital overlap, and spatial confinement of atomic sites, thereby steering CO_2_RR product distributions. For example, polyoxometalate (POM) oxide clusters were used by Zhao et al. [50] as support of single transition-metal atoms (e.g., Sc, Ti, V, Cr, and Mn). Keggin-type Na_4_SiMo_12_O_40_ POMs provided strong metal bonding while allowing significant modification of transition metal d-states. As a result, key intermediates such as *HCOO/*HCOOH were significantly stabilized, leading to highly selective HCOOH or CH_4_ formation with low overpotentials (as low as 0.23 V for Cr). Partially reducible oxides such as TiO_2_ and CeO_2_ have been reported to provide interfacial M–O–Ti or M–O–Ce^3+^ sites via formation of oxygen vacancies [75]. Thus, creation of oxygen vacancies yields interfacial M–O–Ti or M–O–Ce^3+^ sites that strengthen CO_2_ adsorption and facilitate *COOH formation [76]. For instance, CeO_2_-supported M–N_4_-like single sites with adjacent Ce^3+^ centers deliver CO selectivities exceeding 90% at moderate overpotentials, owing to synergistic activation of CO_2_ over both the metal and the oxygen vacancy sites. Similar concepts have been extended to oxyhydroxides, where dynamic lattice oxygen and proton-conducting networks can promote *COOH stabilization and suppress HER, although long-term structural stability under cathodic potentials remains a challenge.

Overall, oxide and oxyhydroxide supports offer several advantages over purely carbonaceous materials. Thus, they provide rich defect chemistry for anchoring and electronically coupling to single atoms. Moreover, oxides such as doped or partially reduced CeO_2_ often enhance CO_2_ uptake through basic surface O^2−^ sites and oxygen vacancies that stabilize carbonate-like species at Ag–O–Ce^3+^ or Cu–O–Ce^3+^ interfaces, thereby improving CO_2_ adsorption at the catalyst–electrolyte interface. Zhou et al. [77] stabilized atomically dispersed Cu^2+^ in a CeO_2_ solid solution, where Cu ions were incorporated into the fluorite lattice of CeO_2_, thus preventing reduction to metallic Cu and aggregation under cathodic CO_2_RR conditions. In this system, neighboring Ce^3+^/oxygen-vacancy sites around the Cu centers strengthened CO_2_ adsorption, lowering the barriers for *CO and *CHO formation, and thus favoring the subsequent hydrogenation steps required for the eight-electron pathway to CH_4_ at relatively low overpotentials and with good stability. In a related study, Liang et al. [76] anchored isolated Ag atoms on CeO_2_ by creating interfacial Ag–O–Ce^3+^ sites associated with oxygen vacancies. Operando spectroscopy and DFT revealed these sites to promote the formation of surface carbonate and *COOH intermediates at the vacancy-rich ceria surface, thereby increasing CO production with FEs above 95% over a wide potential window. On the other hand, the relatively low electronic conductivity of oxide and related materials represents an important issue. Thus, these materials typically require nanostructuring or hybridization with conductive carbons to achieve moderate current densities [6].

### 4.3. MXenes

MXenes have rapidly emerged as versatile supports for SACs in CO_2_RR owing to their unique combination of high conductivity, tunable surface terminations, and strong metal–support interactions. Across theory and experiment, the literature shows that MXenes can stabilize isolated metal centers, modulate their electronic structure, and steer CO_2_RR selectivity [78,79]. Most mechanistic studies on into MXene-SAC for CO_2_RR are purely theoretical, mostly based on DFT. Athawale et al. [80] investigated on transition-metal (TM) SACs supported on O-terminated Ti_2_CO_2_ MXenes. These authors found that isolated metals at hollow-C or bridge sites have the ability to substantially activate CO_2_ and lower the barriers for the formation of *COOH and the transformation of *CO into C_1_ products. Interestingly, activity was found to be related to the energies and occupancy of the metal d-orbitals and Bader charge of the SAC, identifying Ti@Ti_2_CO_2_ as a particularly promising catalyst with low overpotentials and suppressed HER compared with other transition-metal SACs. In a complementary high-throughput study, Cao et al. [81] tested a large number of transition metals on Ti_3_C_2_O_2_ with the aim to mimic M–N_4_-like environments of carbon supports. The authors found that Ru–NS–Ti_3_C_2_O_2_ can reduce activation energies for *COOH and *CO formation to a point in which CO can be theoretically produced with low overpotentials and good selectivity. Vidal-Lopez et al. [82] compared M/Mo_2_CO_x_ and M/Ti_2_CO_x_ (M = Fe, Ru, Co, Rh, Ni, Pd, Pt, Cu, Ag, Au) in terms of CO_2_ activation barriers and bond-breaking energetics. CO_2_ activation was found to follow a two-step mechanism, where CO_2_ first binds at the M–MXene interface and cleaves into *CO and *O. Cu, Ni, Rh, and Pt on Mo_2_CO_x_ and Cu, Ru, and Rh on Ti_2_CO_x_ were identified as the metals with the lowest CO_2_-cleavage barriers, highlighting a strong dependence on both the metal and the support. Al-Mahayni et al. [83] used DFT to test ten metals on four MXene supports with particular emphasis on C_1_ products with highly electron transfer (e.g., methane and methanol). Ni@Ti_3_C_2_O_2_, Pd@Ti_3_C_2_O_2_, and Ru/Fe/Co@Mo_2_CO_2_ were found to have potential for methanol production, while Ni@Ti_3_C_2_O_2_ showed an exceptionally low reaction energy of 0.27 eV for methane. The study concludes that these SACs anchored on MXene can simultaneously suppress HER and C–C coupling, enabling selective CO_2_ conversion to difficult to obtain C_1_ products although these predictions still require experimental validation under realistic CO_2_RR conditions to assess their true activity and stability.

As indicated above, experimental works on SACS supported on MXenes are scarce in the literature. Krishnan et al. [84] investigated two-dimensional Ti_3_C_2_T_x_ MXene nanosheets (without single-atom metal sites) and showed that they catalyze the CO2RR in aqueous electrolyte with a product distribution of CO (42.2%), methanol (23.6%), ethanol (20.1%), and acetone (10.1%) while maintaining stable performance over 72 h. This work demonstrates that conductive pristine Ti_3_C_2_T_x_ alone can deliver mixed C_1_/C_2_^+^ products and serves as a useful baseline for evaluating MXene-supported SACs. Complementarily, Bao et al. [85] anchored isolated Cu single atoms on Ti_3_C_2_T_x_ for electrochemical CO reduction, achieving 98% overall selectivity to multicarbon products with a FE of 71% for ethylene at −0.7 V vs. RHE, stable for 68 h. DFT calculations revealed atomically dispersed Cu–O_3_ sites to favor *CO–*CO coupling. Although the feedstock is CO rather than CO_2_, this study provides direct evidence that MXene-anchored single Cu sites can efficiently promote C–C coupling during CO_2_RR.

## 5. Perspectives and Future Outlook

The advances discussed in this review show that metal–support interactions in SACs have already transformed CO_2_RR to CO into a mature, high-performing reaction. Industrially relevant current densities and nearly 100% selectivities to CO are clear indicatives that this pathway is well developed and ready to be scale up. However, the generation of more reduced C_1_ (e.g., CH_4_, methanol) and C_2_^+^ (e.g., ethanol, acetone) products, the full understanding of the catalytic pathways leading to these products, and the demonstration of technological viability still remain unsolved. Based on the mechanistic and materials concepts described herein, we believe that the following research directions are important in order to advance the CO_2_RR on SAC to more appealing multielectron products.

As indicated above, current SAC technologies excels at the two-electron conversion of CO_2_ to CO, whereas it struggles when multiple proton–electron transfers and C–C coupling steps are required, which explains the much lower FEs typically observed for methanol, methane and C_2_^+^ products. In this sense, moving beyond CO will require new metal–support architectures that simultaneously stabilize key *CO-derived intermediates (e.g., *CHO, *CH_2_O, *OCH_3_,) while preventing site poisoning by strongly adsorbed *CO or oxygenated fragments. This delicate balance, however, can be achieved by playing with the strong effect of the coordination environment and electronic structure of isolated metal atoms on the electrocatalytic performance of the atomic metal site. In this sense, three complementary strategies can be used from the descriptors and design principles described herein: (i) shifting the CO adsorption energy of the single sites into the “intermediate” regime through second-sphere doping or curvature engineering of the support, thereby enabling further *CO hydrogenation instead of premature desorption; (ii) promoting dual-site architectures by preparing single atoms or atom–cluster ensembles embedded in confinement domains, thereby increasing the surface coverage of *CO/*CHO required for C–C coupling without sacrificing the benefits of atomic dispersion; and (iii) exploiting microenvironment engineering (e.g., hydrophobic channels, local alkalinity, tailored cation solvation) to accumulate CO in the vicinity of SAC sites such that the formation of *CHO intermediates is favored at lower overpotentials.

Operando characterization of dynamic single sites will be of high importance to advance towards a better understanding of the catalytic pathways. Thus, as recently revealed [86], the active state of SACs during CO_2_RR is often significantly different from that obtained by ex situ techniques owing to potential-induced restructuring, changes in coordination, and reversible aggregation/dissolution processes. Conventional post-reaction microscopy is clearly insufficient to capture these transformations, especially under the high current densities and mass-transport regimes relevant to practical devices. Thus, systematic deployment of operando techniques based on X-ray absorption spectroscopy (to follow oxidation state and coordination numbers), Raman and infrared spectroscopies (to track adsorbed intermediates), and ambient-pressure XPS (to study interfacial speciation and electrolyte-induced restructuring under realistic gas-electrolyte conditions) are crucial. Remarkably, the growing complexity of multi-component SAC systems will soon need data-driven analysis of these operando datasets in order to obtain reliable results. Thus, recent studies combining time-resolved XANES with machine learning illustrate how Ni–N_x_ sites evolve during CO_2_ reduction [87]. This outstanding and paradigmatic example provides an approach that can be extended to other metals, supports and products. The final goal is to correlate the real-time population of specific atomic configurations and adsorbate states with instantaneous activity and selectivity metrics, thereby converting mechanistic insights into predictive rules for catalyst and reactor design.

Throughout this review, DFT has been proposed as a valid tool to rationalize scaling relationships, d-band descriptors, and microenvironment effects in CO_2_RR over SACs. However, systems containing the metal site, first-shell ligands, second-sphere dopants, curvature and defect motifs are too complex to be explored by DFT. As has been recently demonstrated by Wen et al. [88], integrating machine learning with DFT offers a route to accelerate this exploration. For example, high-throughput computations [81] on representative metal-ligand sites anchored on different supports can be used to train numerical models that map easily accessible structural features (e.g., coordination number, Bader charge, projected density of states, local curvature, hydrophobicity indicators) onto key thermodynamic descriptors (Gibbs energy for key intermediates). Once validated against experiments, these models could help screen thousands of hypothetical SAC–support combinations for targeted product windows (e.g., CO, formate, methanol, methane, C_2_^+^) under specified potential and pH conditions, thereby narrowing down the synthetic search to a shortlist of high-value candidates. It is also necessary to point out that despite their usefulness, current DFT approaches for SACs in CO_2_RR present important methodological limitations that must be acknowledged. Predicted adsorption energies and reaction pathways are often sensitive to the choice of exchange–correlation functional and dispersion treatment, and to whether solvation is described implicitly or with explicit water, which can lead to non-negligible variations in limiting potentials and reaction barriers. In addition, dynamic coordination changes, multiple spin states, and charged intermediates at single-atom sites are difficult to capture accurately, while standard constant-charge slab models provide only an approximate description of electrode potential and electrolyte effects. Consequently, there can be quantitative discrepancies between computed energetics and experimental onset potentials or selectivity windows, and DFT-derived descriptors and volcano plots should therefore be interpreted primarily as qualitative trends and, whenever possible, validated by operando spectroscopy and experimental data.

While the synthetic routes described in the current SAC literature (e.g., MOF-derived carbons, high-temperature pyrolysis, acid leaching, or elaborate atomic layer deposition protocols) are well suited for mechanistic studies, they are far from being scalable and cost-effective [89,90]. Future work must therefore address scalability at both the catalyst and the device level by designing new scalable, low-waste techniques such as mechanochemistry, which can deliver SACs in gram to kilogram-scale batches while minimizing solvent use and processing steps. Mechanochemical ball-milling routes allow to disperse isolated metals on oxide and carbon supports under ambient conditions, and recent reviews have highlighted their potential to produce SAC across different metals and supports [91,92]. As rightly indicated by Sun and co-workers [93], techno-economic analyses including specific aspects of SACs (e.g., metal loading, support processing, stability and replacement intervals, product separation costs for multi-product mixtures) are crucial to identify realistic product targets. Thus, while CO and formate are already close to feasibility [94], multielectron products such as methanol, ethanol or higher hydrocarbons will likely require significant improvements in terms of FE, current density, and long-term stability before they can compete with thermocatalytic or thermochemical routes [95].

## 6. Conclusions

The studies discussed in this review demonstrate that metal–support interactions are a key tool to control the activity, selectivity and durability of SACs during CO_2_RR. Thus, by tailoring the coordination environment of isolated metal centers through heteroatom doping, vacancy engineering, curvature, and surface functionalization of the support, it is possible to modulate the adsorption energies of crucial intermediates (such as *COOH, *OCHO, *CO and *CHO) and steer the reaction towards specific C_1_ or C_2_^+^ products while suppressing parasitic reactions such as HER. In particular, pyridinic-type M–N_4_ sites on N-doped carbons, curvature-tuned graphene and carbon nanotubes, and defect-rich porous carbons have emerged as robust supports to reach nearly quantitative CO selectivity at industrially relevant current densities. Non-carbon supports such as oxides, polyoxometalates, MXenes, MOFs, and zeolites can also provide new ways of tuning metal–support interactions. This review stressed that quantitative descriptors such as d-band position, projected density of states, and local electric field can be used as useful tools to rationalize performance trends and to guide the design of new supports and coordination architecture.

Despite the rapid development of this field, several challenges must be addressed before SACs can reach industrially relevant performance. The mechanistic pathway for methanol, methane and C_2_^+^ formation on isolated sites remains unsolved. Comprehensive operando studies that combine structural and spectroscopic techniques with advanced data analysis will be crucial to study the actual active states and to connect their evolution with the activity and selectivity in real time. In parallel, integrating machine learning with atomistic calculations and experimental descriptors is expected to accelerate this exploration.

From a technological standpoint, synthetic routes widely used today are not yet fully compatible with the requirements of large-scale. Emerging mechanochemical methods provide promising pathways for scalable production of SACs with controlled coordination and loading. Techno-economic analyses indicate that CO and formate are currently the most realistic targets, whereas more reduced products will require substantial gains in FE, current density, and operational stability.

## Data Availability

No new data were created or analyzed in this study.
